# Masseter Muscle Myofibrillar Protein Synthesis and Degradation in an Experimental Critical Illness Myopathy Model

**DOI:** 10.1371/journal.pone.0092622

**Published:** 2014-04-04

**Authors:** Hazem Akkad, Rebeca Corpeno, Lars Larsson

**Affiliations:** 1 Department of Neuroscience, Clinical Neurophysiology, Uppsala University, Uppsala, Sweden; 2 Department of Biobehavioral Health, The Pennsylvania State University, University Park, Pennsylvania, United States of America; Institut de Myologie, France

## Abstract

Critical illness myopathy (CIM) is a debilitating common consequence of modern intensive care, characterized by severe muscle wasting, weakness and a decreased myosin/actin (M/A) ratio. Limb/trunk muscles are primarily affected by this myopathy while cranial nerve innervated muscles are spared or less affected, but the mechanisms underlying these muscle-specific differences remain unknown. In this time-resolved study, the cranial nerve innervated masseter muscle was studied in a unique experimental rat intensive care unit (ICU) model, where animals were exposed to sedation, neuromuscular blockade (NMB), mechanical ventilation, and immobilization for durations varying between 6 h and 14d. Gel electrophoresis, immunoblotting, RT-PCR and morphological staining techniques were used to analyze M/A ratios, myofiber size, synthesis and degradation of myofibrillar proteins, and levels of heat shock proteins (HSPs). Results obtained in the masseter muscle were compared with previous observations in experimental and clinical studies of limb muscles. Significant muscle-specific differences were observed, i.e., in the masseter, the decline in M/A ratio and muscle fiber size was small and delayed. Furthermore, transcriptional regulation of myosin and actin synthesis was maintained, and Akt phosphorylation was only briefly reduced. In studied degradation pathways, only mRNA, but not protein levels of MuRF1, atrogin-1 and the autophagy marker LC3b were activated by the ICU condition. The matrix metalloproteinase MMP-2 was inhibited and protective HSPs were up-regulated early. These results confirm that the cranial nerve innervated masticatory muscles is less affected by the ICU-stress response than limb muscles, in accordance with clinical observation in ICU patients with CIM, supporting the model' credibility as a valid CIM model.

## Introduction

Acute quadriplegic myopathy, or more commonly named critical illness myopathy (CIM), is the most common acquired muscle wasting and weakness in immobilized and mechanically ventilated intensive care unit (ICU) patients [1]. CIM is characterized by a preferential loss of myosin and myosin-associated proteins while thin-filament proteins are spared or less affected [1]–[3]. Axial (limb and trunk) skeletal muscles are usually strongly affected by CIM, while craniofacial muscles are spared or less affected [1], [2]. Muscle wasting and dysfunction develop after one week exposure to ICU treatment [2], [4], [5] and can extend to the respiratory muscles, rendering the patient ventilator-dependent [4]–[7]. CIM has significant negative effects on morbidity, mortality, quality of life and health care costs generated by CIM complications and the following rehabilitation [8]–[11]. CIM has been suggested to be a consequence of modern critical care treatments, including immobilization, mechanical ventilation, systemic administration of corticosteroids, post-synaptic neuromuscular blockade (NMB), and sepsis [1], [2], [12], but the underlying mechanisms and the temporal unfolding of pathological events are less clear.

The study of CIM patients is typically complicated by multiple factors, such as variations in patient age, concomitant disorders and interventions, etc. Consequently, there is a significant need for an experimental model where these confounding factors are eliminated. Based on these premises, our group has used rodent and porcine experimental ICU models in parallel with clinical research to generate a plethora of useful data on CIM underlying mechanisms [13]–[24]. By studying protein synthesis and degradation pathways in skeletal muscles, our previous studies have shown that mechanical silencing *per se* (lack of mechanical loading associated with muscle contraction and weight bearing) is an important trigger of CIM. In the rodent model, a phenotype considered pathognomonic of CIM in ICU patients was observed, i.e., severe muscle wasting, weakness, and a preferential myosin loss in limb muscles [13]–[16], [22]–[24], but most of these studies focused on spinal nerve innervated muscles, such as limb and respiratory muscles.

The current time-resolved study aims at enhancing our understanding of mechanisms of the relative sparing of a cranial nerve innervated muscle (the masseter) in response to mechanical silencing, in comparison to spinal nerve innervated muscles studied previously. We used the experimental rat model in time-resolved analyses with high temporal resolution from six hours to two weeks. The experimental model includes sedation, post-synaptic NMB, immobilization, and mechanical ventilation. Several muscular atrophy hallmarks were subsequently analyzed, including muscle fiber size, myosin to actin (M/A) ratio, transcriptional regulation and protein expression of myosin and actin, the protein synthesis inducer Akt, the proteolytic pathways of atrogenes (MuRF1 and atrogin-1), autophagy and metalloproteinase and finally, the protective heat shock proteins (HSPs). Results from this study are compared with previous results from limb muscles obtained from the same experimental ICU model and other unloading animal models, allowing further evaluation of the model's credibility as a CIM model. We hypothesize that cranial nerve innervated masticatory muscles respond differently from limb muscles and employ protective mechanisms to curb proteolysis and sustain sarcomeric protein synthesis; hence, allowing prolonged sparing of thick filament proteins and muscle fiber size.

## Materials and Methods

### Animals

Sixteen female Sprague–Dawley rats were divided into four groups, i.e., four animals per group; one control group and three experimental 0.25–4, 5–8 and 9–14 day groups. For comparison, the same duration intervals were chosen as in our previous studies limb muscles [13], [15]. Except for the group of control rats, the 3 experimental groups were mechanically ventilated and treated with α-cobratoxin and isoflurane, a non-depolarizing neuromuscular blocker and an inhalational anesthetic, for durations of 0.25–4, 5–8 and 9–14 days, respectively. The control rats were sham-operated and underwent the same interventions as the experimental rats, but without the administration of α-cobratoxin. That is, control rats were anaesthetized with isoflurane, spontaneously breathing, given intra-arterial and intra-venous solutions (*see below*), and euthanized within 2 h after the initial anaesthesia and surgery.

The experimental model has been fully described elsewhere [25], [26]. In brief, all the following surgeries and instrumentation were performed with sterile technique: (1) precordial silver wire electrocardiogram (ECG) electrodes were implanted subcutaneously. (2) An aortic catheter (28-gauge Teflon) was inserted via the left carotid artery to record arterial blood pressure. (3) For parental solutions, a 0.9 mm Renathane catheter was threaded into the left jugular vein. 4) Two subcutaneous electroencephalogram (EEG) needle electrodes were placed into the skull above the right and left temporal lobes, and a third reference electrode was placed in the neck region. (5) A 37°C servo-regulated vaginal thermistor for measuring temperature. (6) A silicone cannula was inserted in the urethra for continuously recording urine output. For protein and fluid balance, the administration of the following solutions was maintained: (1) an intra-arterial solution (0.6 ml·h^−1^) consisting of 50 ml H_2_O, 50 ml 0.5 N lactated Ringer solution, 1.25 g oxacillin sodium, 2.8 mg *α*-cobratoxin, and 20 meq K^+^ (as KCl); and (2) an intra-venous solution (0.6 ml·h^−1^) consisting of 50 ml H_2_O, 50 ml 0.5 N lactated Ringer solution, 12.8 ml 50% glucose (Fresenius Kabi, Uppsala, Sweden) and 1.25 g oxacillin sodium.

The levels of the anaesthetic isoflurane were maintained at >1.5% during the surgery or any possible irritating manipulation, maintaining the following condition: (1) synchronized EEG and dominated by high-voltage slow-wave activity; (2) mean arterial pressure at 100 mmHg and heart rate at 420 beats·min^−1^; and, 3) absence of evident responses of EEG, blood pressure and heart rate to surgical procedures. Isoflurane was delivered into the inspiratory gas stream by a precision mass-flow controller. After the initial surgery, levels of isoflurane were gradually decreased over 1–2 days and maintained at <0.5% during the rest of the experimental period. Rats were ventilated via a coaxial tracheal cannula at 72 breaths·min^−1^ with an inspiratory:expiratory ratio of 1∶2. A minute volume of 180–200 ml and gas concentrations of 49.5% O2, 47% N2 and 3% CO2 was delivered by a precision volumetric respirator (volume drift <1% per week). Intermittent hyperinflations (6 per hour at 15 cmH_2_O), positive end-expiratory pressure (PEEP) (1.5 cmH_2_O), and expiratory CO_2_ monitoring were continuous. NMB was induced on the first day (100 μg I.V. *α*-cobratoxin) and maintained by continuous infusion (250 μg·day^−1^, I.V.). Mechanical ventilation started immediately after the NMB induction. Experiments were terminated at durations varying between 6 h and 14 days. The animals displayed no signs of infections or septicaemia throughout the whole experiments. The Institutional Animal Care and Use Committee at the Pennsylvania State University College of Medicine and the Ethical committee at Uppsala University approved all aspects of this study.

### Sodium dodecyl sulphate-polyacrylamide gel electrophoresis (SDS-PAGE)

Total protein content was determined using 10-μm cross-sections from the masseter muscle dissolved in 100 μl 8 M urea buffer after centrifugation and heating (90°C for 2 min), using the Pierce 660 nm Protein Assembly Assay reagent (Thermo Scientific, Rockford, USA). The absorbance of the samples was measured using a plate reader Multiskan Ex, with the software Ascent iEMS Reader MF, version 2.6 (Thermo Scientific) and related to a standard curve of bovine serum albumin (Thermo Scientific) at concentrations ranging from 250 μg ml^−1^ to 1500 μg ml^−1^.

To prepare the masseter muscle samples for the subsequent electrophoresis analyses, six 10-μm cross-sections of the masseter were dissolved in urea buffer (120 g urea, 38 g thiourea, 2.89 g dithiothreitol, 1.51 g Trizma base, 7.5 g sodium dodecyl sulfate (SDS) and 0.004% bromophenol blue, H_2_O up to 250 ml) and a volume of 5 μl was loaded on sodium dodecyl sulfate-polyacrylamide gel electrophoresis (SDS-PAGE) 6% for determining the composition of myosin isoforms, and 12% for measuring the myosin/actin ratio. The total acrylamide and Bis concentrations were 4% (w/v) in the stacking gel and 6% or 12% (w/v) in the running gel, for the respective gels. The gel matrix included 30% and 10% glycerol in the 6% and 12% gels, respectively. Briefly, the 6% gel electrophoresis was performed at a constant voltage of 120 V for 20–22 hours at 10°C while the 12% gel electrophoresis was performed at a constant current of 16 mA for 5 h at 15°C. Tris–glycine electrode buffer (pH 8.3) (SE 600 vertical slab gel unit, Hoefer Scientific Instruments, San Francisco, CA, USA) was used with both gels.

The 6% gels were silver-stained, whereas 12% SDS-PAGE gels were stained with Coomassie blue (SimplyBlue SafeStain Invitrogen). Soft laser densitometer (Molecular Dynamics, Sunnyvale, CA, USA) was used to scan all the gels with a high spatial resolution (50 μm pixel spacing) and 4,096 optical density levels to determine the relative contents of myosin heavy chain (MyHC) isoforms. The volume integration function (ImageQuant TL Software v. 2003.01, Amersham Biosciences, Uppsala, Sweden) was used for quantification of protein content on the gels.

### Hematoxylin and eosin (H&E) and cross-sectional area measurements

Cross-sections (10 μm) were cut perpendicular to the muscles greatest girth in a cryostat at −20°C. The cross-sections were incubated in hematoxylin solution (HHS16, Sigma-Aldrich, Munich, Germany) for 2 minutes, rinsed with distilled water, incubated in eosin solution (HT-110-1-16, Sigma-Aldrich, Munich, Germany) for 2 minutes and finally rinsed with 95% ethanol and mounted on an inverted microscope (Axiovert 40 CFL, Carl Zeiss Microscopy, USA) connected to a CCD camera (VCC-2972, SANYO Electric Co.). The cross-sectional area (CSA) and the lesser diameter of 70–100 muscle fibers, regardless of fiber types, was measured semi-automatically on H&E stained section with the aid of an imaging application (Simple PCI 6.0 Hamamatsu Corporation, USA).

### Immunoblotting

Five μg of total protein of the masseter samples were loaded per lane and run on the SDS-PAGE using Mini-PROTEAN 3 Cell (Bio-Rad Laboratories, 2000 Alfred Nobel drive, Hercules, CA, USA) at constant 120 volts for 90 minutes. Acrylamide concentrations were 4% and 12% (w/v) in stacking and running gels, respectively, and the gel matrix included 10% glycerol. Protein blots were subsequently transferred from the gels to polyvinylidene fluoride Immobilon Transfer membranes (Millipore, Billerica, USA) at 350 mA current for 90 minutes using Bio-Rad KIT.

Membranes were incubated with primary antibodies of MuRF1 (AF5366, R&D Systems, MN, USA), atrogin-1 (AP2041 ECM Biosciences, KY, USA), HSP70, HSP90 and αB-crystallin (SMC-100B, SMC-137C and SMC-159A, respectively, StressMarq Biosciences Inc. Canada), LC3b (L754, Sigma-Aldrich, Munich, Germany), Akt and p-AKt (9272, 9271 respectively, Cell Signaling Technology, Inc., Danvers, Canada), MMP-2 and TIMP-2 (MAB3308, CC1064, respectively, Merck Millipore, USA), and actin (sc-1616-R, Santa Cruz Biotechnology Inc., CA, USA).

The membranes were incubated with secondary antibodies (NA934, GE Healthcare) or (sc-2020, Santa Cruz Biotechnology Inc.). ECL Advance Western blotting detection kit (RPN 2135, Amersham Biosciences) and Odyssey Imaging Systems (LI-COR Biosciences UK Ltd) were used for two-color infrared fluorescent detection according to manufacturer's instructions. The intensity volumes of each protein signal were normalized to intensity volumes of actin.

### Quantitative real-time PCR

The RNA for quantitative real-time polymerase chain reaction (RT-PCR) was extracted from frozen muscle tissue (10–30 mg) using a Qiagen RNeasy Mini Kit (Qiagen, Valencia, CA, USA) as follows: (1) muscle tissue was homogenised using a rotor homogeniser (Eurostar Digital, IKA-Werke), (2) QIAshredder Columns (Qiagen, Valencia, CA, USA) were used to disrupt DNA, and (3) total RNA was eluted from RNeasy Mini columns with 30 μl of RNase-free water. The RNA concentrations were quantified afterwards using the fluorescent nucleic acid stain, Ribogreen (Molecular Probes, Eugene, OR, USA) on a Hitachi F-4000 fluorescence spectrophotometer. Total RNA (100 ng) was reverse-transcribed to cDNA using Qscript cDNA Supermix (Quanta Biosciences, USA) and the cDNA was diluted to a volume of 100 μl and stored at −80°C until real-time PCR quantification.

Quantitative real-time PCR was used to quantify the mRNA levels of MyHC (types IIx and IIb), skeletal α-actin, atrogin-1, MuRF1, and ribosomal RNA 18S was used as an internal standard (GenBank accession numbers AF157005, L24897, NM019212, AY059628, AY059627, AF102857, respectively). Taqman primers (Table 1) were designed using the software Primer Express (Applied Biosystems, Foster City, CA, USA). The primers were purchased from Thermo Electron (Thermo Electron, Ulm, Germany) and purified by high-performance liquid chromatography. SYBR Green (1988123, Roche Diagnostics, Germany) was used for detection of all the studied genes. Complementary DNA was amplified using MyiQ single colour real-time PCR detection system (Bio-Rad Laboratories, Inc., Hercules CA, USA). The AmpliTaq Gold DNA polymerase was heat-activated at 95°C for 10 min, followed by 50 cycles of a two-step PCR with denaturation at 95°C for 15 seconds and a combined annealing and extension step at 60°C for 1 minute. The PCR was performed in a volume of 25 μl, which included 0.4 μM of each primer and 0.2 μM of SYBR Green.

**Table 1:** Primers and probes used in real time PCR analyses.

Gene	Forward primer sequence	Reverse primer sequence	Amplicon length
Myosin heavy chain IIb	GAGCTTGAAAACGAGGTGGAAA	TGCTTGCGAAGACCCTTGA	68
Myosin heavy chain IIx	TCGCCGAGTCCCAGGTC	CGCTTATGATTTTGGTGTGAACC	66
α-Actin	AGGTCATCACCATCGGCAAT	AAGGAAGGCTGGAAGAGCGT	61
atrogin-1	TCCTGGATTCCAGAAGATTCAAC	TCAGGGATGTGAGCTGTGACTT	75
MuRF1	ACAACCTCTGCCGGAAGTGT	CCGCGGTTGGTCCAGTAG	67
LC3b	CTCATCCGGGAGCAGCAT	CTCACCCTTGTATCGCTCTATAATCA	60
18S	GTGCATGGCCGTTCTTAGTTG	AGCATGCCGAGAGTCTCGTT	74

Each sample was run in triplicates. With each PCR run, a standard cDNA was included in triplicates of three concentrations comprising a standard curve. A control sample was used for the standard and a non-template control (NTC) was included on each plate. The threshold cycle (CT) data acquired from the real-time PCR run was related to the standard curve to obtain the starting quantity (SQ) of the template cDNA for each sample. Each sample in a triplicate had to be within 0.5 CT of each other to be included in the analysis. The triplicates of each sample were averaged and the SQ of the sample was related to the triplicate average of the internal standard, 18S. To be accepted, the slopes of the standard curves had to be between −3.0 and −3.5 and were not allowed to differ by more than 5%. The values of the samples, related to the standard, were then analysed.

### Statistics

Means and standard errors of the means (SEM) were calculated according to standard procedures. One-way analysis of variance (ANOVA) and the Tukey *post hoc* test were used for comparing multiple groups and *p*<0.05 was considered statistically significant.

## Results

### Muscle fiber size

**Figure 1:**
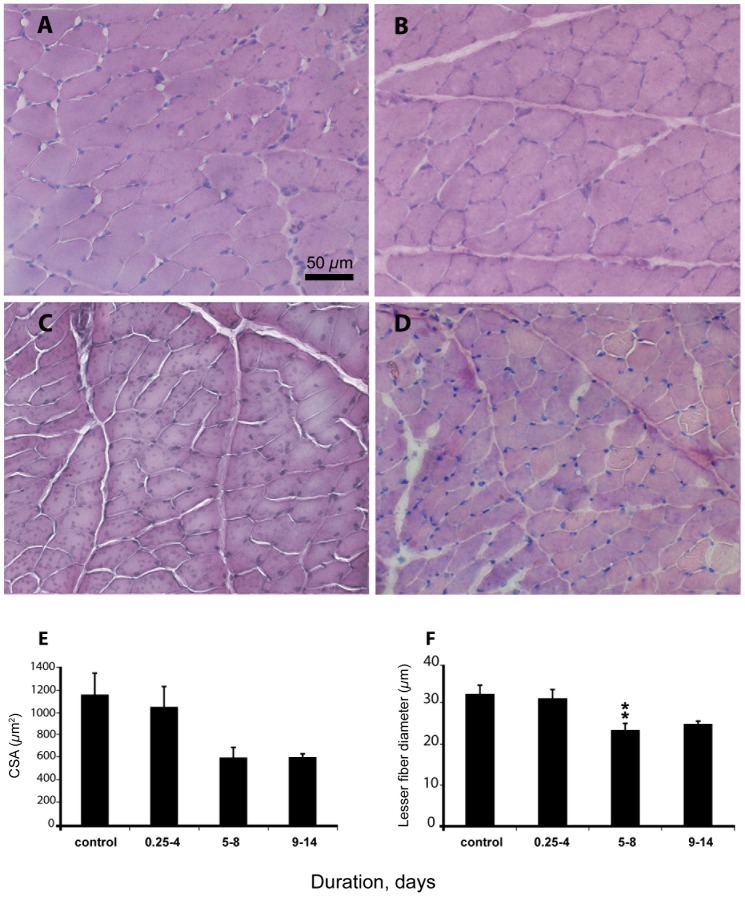
Hematoxylin and eosin (H&E) staining and cross-sectional area (CSA). **A, B, C, D**) H&E staining for the measurement of CSA of 0.25–4, 5–8 and 9–14 day groups, respectively, **E**) CSA (μm^2^) of the masseter muscle fibers, and **F**) the lesser fiber diameter (μm). Asterisks (**p<0.01) denote significant differences compared with controls. Values are means + SEM.

Muscle fiber size was measured both as cross-sectional area (CSA) and as the lesser fiber diameter. CSA measurements often overestimate fiber size due to muscle fibers being obliquely cut, an error that cannot be completely ruled out in muscle cross sections. The lesser fiber diameter, on the other hand, is not affected by this error as long as the cut passes the center of the fiber [27]. According to one-way ANOVA, the ICU condition had a significant negative effect on masseter muscle fiber size measured as both CSA (p<0.05) and the lesser fiber diameter (p<0.01), but *post hoc* Tukey analysis identified a significant decline only in the lesser fiber diameter in the 5–8 day group (Figure 1).

### Myosin/actin ratios

**Figure 2:**
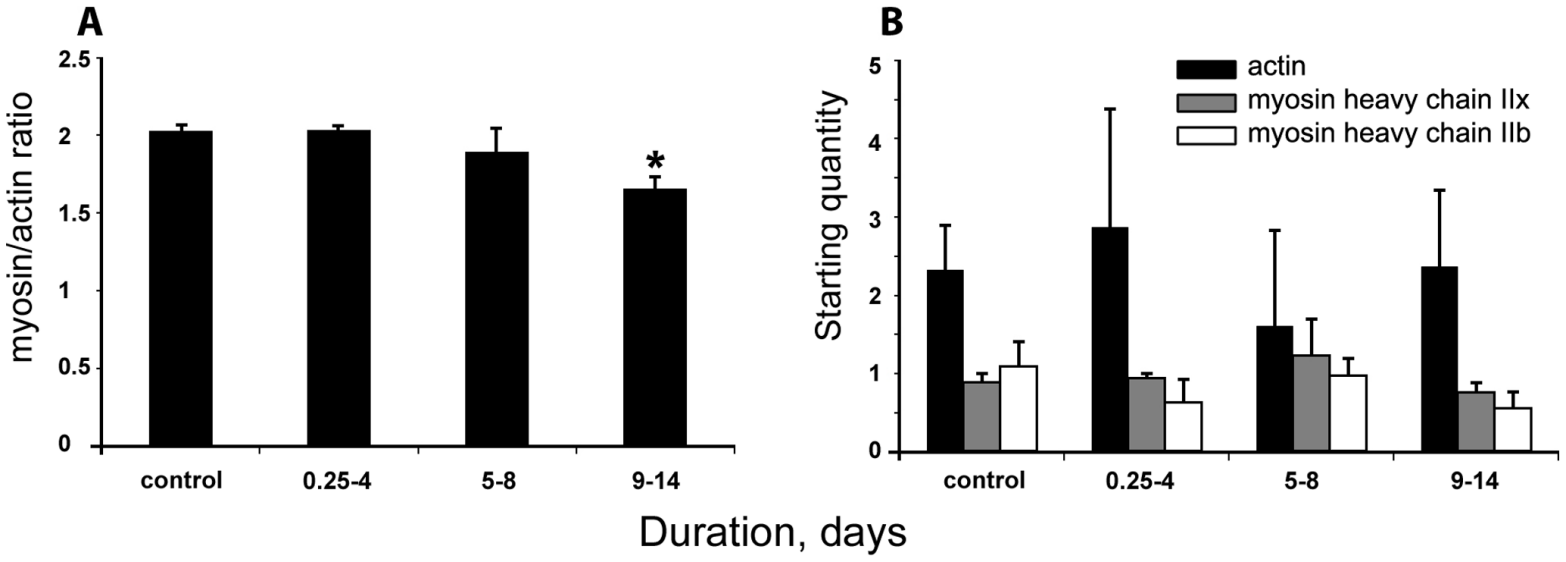
Myosin/actin protein ratios, myosin and actin mRNA expression. **A**) myosin/actin ratios determined by 12% SDS PAGE. Significant difference compared with controls and 0.25-4 day group is indicated with an asterisk (*p<0.01). **B**) mRNA of alpha-actin (black bars) and myosin heavy chain (type IIx: grey bars and type IIb: open bars). No significant differences were observed in myofibrillar protein mRNA expression. Except for the control group, all rats were exposed to immobilization, mechanical ventilation, post-synaptic neuromuscular blockade for 0.25–4, 5–8 and 9–14 days. N = 4 per group. Values are means + SEM.

Only two myosin heavy chain (MyHC) isoforms were observed in the masseter according to sensitive silver stained 6% SDS PAGE, i.e., the fast IIx and IIb MyHC isoforms were expressed in almost equal proportions. The 2∶1 stoichiometric relationship between myosin and actin content was well-preserved until the longest duration (9–14 days) where the M/A ratio declined (p<0.01) by 15% compared with controls and 0.25–4 day groups (Figure 2A).

### Myosin and actin mRNA expression

The mRNA expression of the dominant thick filament proteins in the rat masseter, MyHC subtypes IIx and IIb, and actin were measured using RT-PCR. No changes were detected in MyHC or actin mRNA expression during the 14-day observation period (Figure 2B).

### Atrogene and autophagy mRNA expression

**Figure 3:**
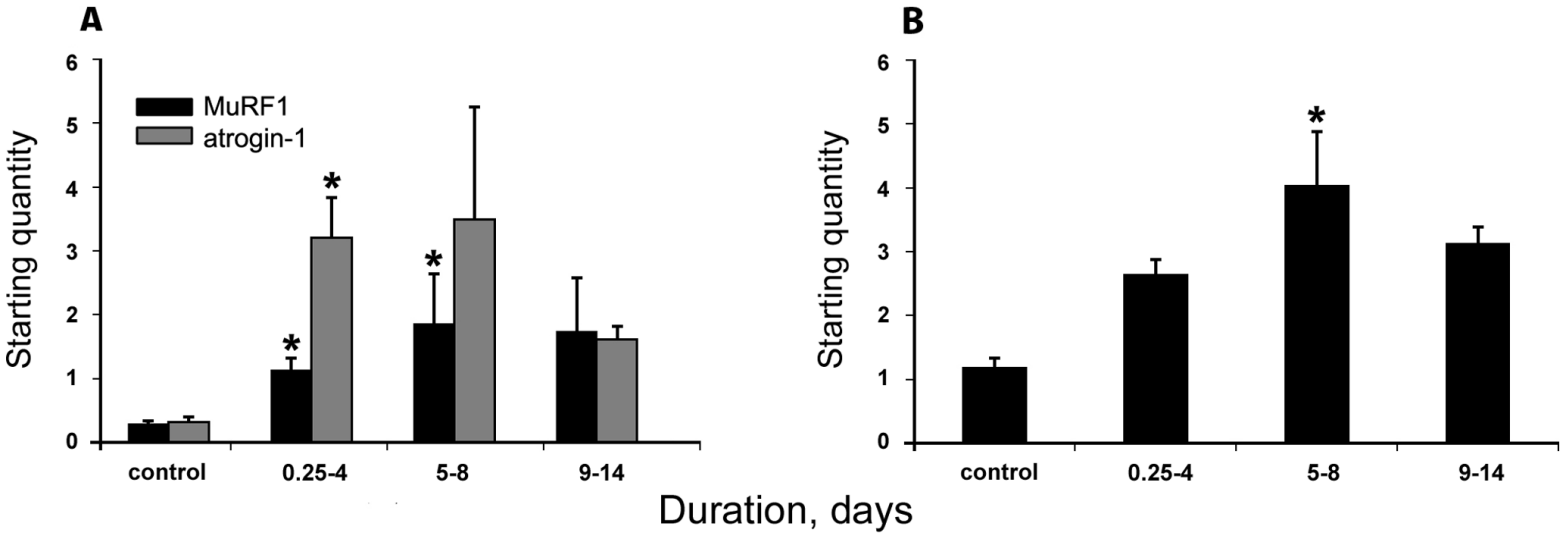
MuRF1, atrogin-1 and LC3b mRNA expression. **A**) MuRF1 (black bars) and atrogin-1 (grey bars), **B**) LC3b mRNA expression, according to real-time PCR in the masseter muscle. Asterisks (*p<0.05) denote significant differences compared with controls. Values are means of starting quantities + SEM.

The ubiquitin proteasome pathway is considered the major proteolytic mechanism in skeletal muscles and plays an important role in limb muscle protein degradation [28], [29]. The transcriptional regulation of the two atrogenes MuRF1 and atrogin-1 showed an initial up-regulation (Figure 3A). MuRF1 mRNA expression showed a rapid significant increase in both the 0.25–4 and 5–8 day groups (p<0.05), but did not differ from controls in the 9–14 day group. Atrogin-1 mRNA expression increased (p<0.05) in the 0.25–4 day group, but did not differ from controls at durations longer than 5 days (Figure 3A). mRNA expression of the autophagy marker, LC3b, showed a gradual increase over time in response to the ICU condition, reaching its maximum in the 5–8 day group (p<0.05, Figure 3B).

### Immunoblotting

Protein levels of the ubiquitin E3 ligases (MuRF1 and atrogin-1) and LC3b were measured in order to validate mRNA expression data. In addition, protein levels of the synthesis-upstream protein kinase (Akt), the phosphorylated activated form of Akt (p-Akt), the collagenase (MMP-2) and its inhibitor (TIMP-2), and three chaperones (αB-crystallin, HSP70 and HSP90) were measured.

**Figure 4:**
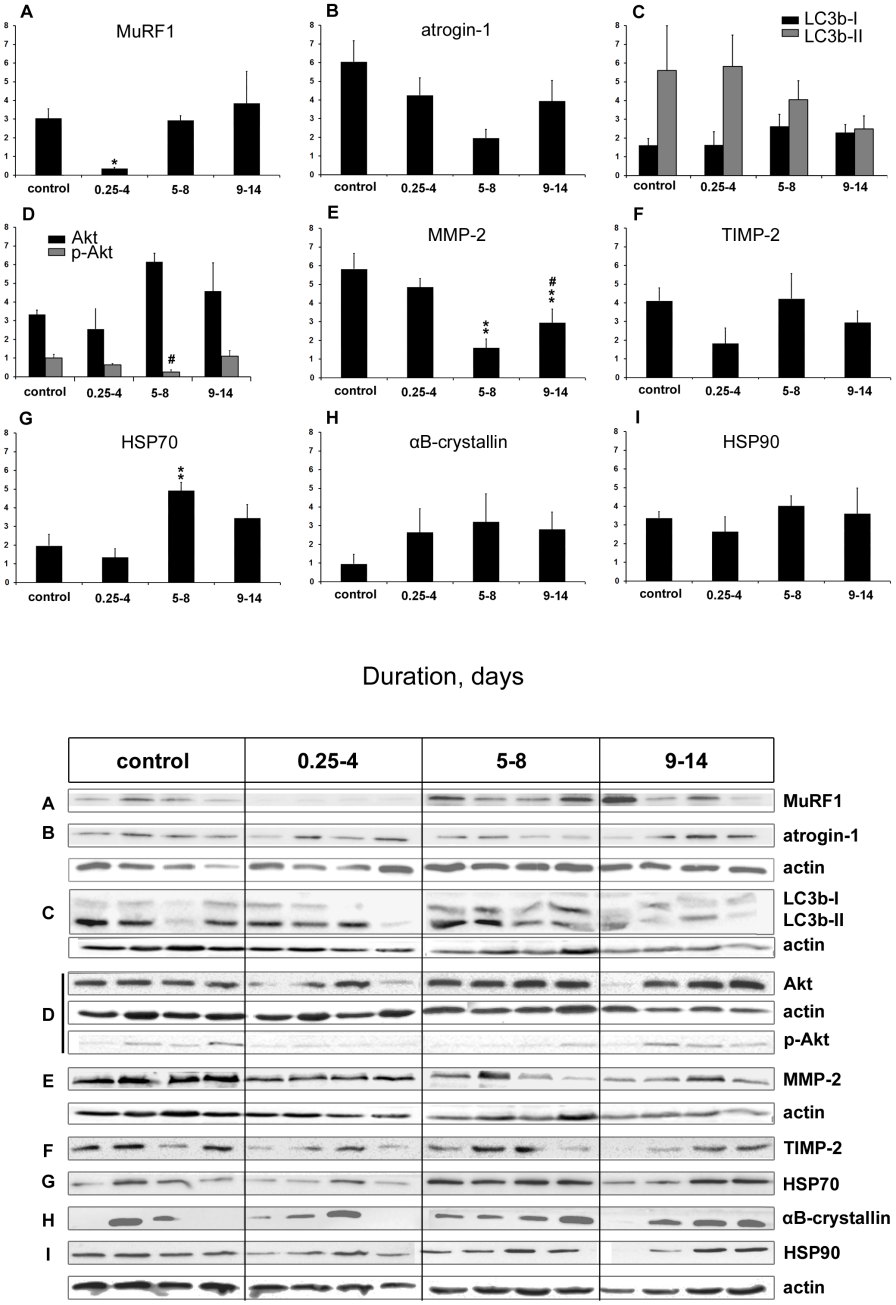
Immunoblotting analyses. Western blot analyses of **A**) MuRF1, **B**) atrogin-1, **C**) LC3b-I (black), LC3b-II (grey), **D**) Akt (black) and phosphorylated Akt (grey), **E**) MMP-2, **F**) TIMP-2, **G**) HSP70, **H**) αB-crystallin, **I**) HSP90. Values are normalized to α-actin content in the masseter muscle in controls and rats exposed to immobilization, mechanical ventilation, post-synaptic neuromuscular blockade for 0.25–4, 5–8 and 9–14 days. N = 4 per group. Asterisk (*p<0.05, **p<0.01) denotes significant difference compared with controls, and hash ^#^p<0.05 denotes significant difference compared with 0.25–4 day group (MMP-2) or 9–14 day group (p-Akt). Values are means of optical intensity (arbitrary units) + SEM.

MuRF1 and atrogin-1 expression at the protein level were not elevated in spite of the early transcriptional up-regulation. MuRF1 protein levels decreased in the 0.25–4 day group, but returned to control levels afterwards. According to one-way ANOVA and *post hoc* Tukey analysis, MuRF1 and atrogin-1 protein levels did not change significantly (Figure 4 A, B, respectively).

With its two isoforms, LC3b is one of the most important autophagy markers and plays an important role in autophagosome membrane biogenesis [30]. Protein levels of both LC3b forms (I and II) showed no difference over time (Figure 4C).

Protein content of Akt was stable throughout the experimental period. However, the phosphorylated active form of Akt, p-Akt, showed a transient (albeit not statistically significant) decline in the 0.25–4 and 5–8 day groups and returned to control values in the 9–14 day group (significantly higher than in the 5–8 day group, Figure 4D).

Protein levels of the matrix metalloproteinase MMP-2 decreased (p<0.01) at the longest durations (5–8 and 9–14 days), but the negative regulator of MMP-2, TIMP-2, did not change during the experimental period (Figure 4E and F, respectively).

The different chaperone proteins responded differently to the ICU condition: HSP70 was significantly increased (p<0.01) in the 5–8 day group (Figure 4G), αB-crystallin showed a trend towards an early and maintained elevated level, albeit not statistically significant (p = 0.06) (Figure 3H), and HSP90 protein levels were not affected by the ICU condition (Figure 4I).

## Discussion

Protein balance in skeletal muscles is a delicate interplay between protein synthesis and degradation [29]. Mechanical ventilation, elimination of weight bearing and lack of neuromuscular activation in ICU patients induce the severe muscle weakness and atrophy known as CIM, characterized by a loss in thick filament proteins. However, while limb and trunk muscles are severely affected by CIM, craniofacial muscles are spared or less affected [1], [2], [4]. In an attempt to unravel underlying mechanisms, an experimental model was used to investigate M/A ratios, muscle fiber size, regulation of myofibrillar protein synthesis at the transcriptional level, different proteolytic pathways and their regulators after exposure to the ICU condition, (i.e., sedation, NMB, immobilization and mechanical ventilation) at durations varying from 6 hours to 14 days in a cranial nerve innervated muscle (the masseter). Significant differences were observed between the masseter and previous observations in limb muscles.

### Myosin/actin ratio, fiber size and protein synthesis

The cranial nerve innervated masseter muscle seems to resist the atrophy-inducing condition for longer periods than distal hindlimb muscles. That is, compared to the corresponding results in hindlimb muscles where M/A ratio and fiber size showed a progressive decline after 5 days of the intervention [13], the M/A ratio decrease in the masseter was smaller and restricted to the longest duration, and muscle fiber size was significantly decreased only in the 5–8 day group. Thus, the rat model confirms the differential masticatory versus limb muscle response to the ICU condition, i.e., the relative sparing of the masticatory muscle in ICU patients with CIM [1], [2], [24].

In the masseter, transcriptional regulation of myofibrillar proteins does not seem to be affected by mechanical ventilation and unloading. mRNA levels of the two most abundant myosin isoforms in the masseter (IIx, IIb) as well as actin were found to be unaffected by the intervention during the two week observation period. In limb muscles, on the other hand, a dramatic transcriptional down-regulation of both myosin and actin was observed at durations longer than five days in the same experimental ICU model [13] as well as in ICU patients with CIM [16], [24].

The highly conserved IGF-1/PI3K/Akt pathway plays an essential role in muscle protein synthesis [31]. The activation of Akt can induce pro-synthesis pathways and is sufficient to block muscle atrophy [32]. Akt has been shown to be activated during hypertrophy and inhibited in hindlimb atrophy models [33]–[36], but studies in humans have casted doubt on the link between Akt signaling and muscle protein synthesis during immobilization [37]. In this study, the briefly down-regulated Akt phosphorylation does not seem to affect myosin and actin synthesis. Another role of Akt is the phosphorylation/inhibition of the nuclear translocation of the FOXO family of transcription factors, thus preventing the up-regulation of atrogenes (MuRF1 and atrogin-1) and protecting thick filament proteins from degradation [38]. This role of Akt was confirmed in the masseter as Akt phosphorylation levels were inversely proportional to mRNA levels of the atrogenes and LC3b, which are regulated by FOXO. The significant increase in Akt re-activation in the 9–14 day group may be explained as a muscle-specific adaptation to stress.

### Protein degradation pathways

Different protein degradation pathways may be involved in sarcomeric protein loss. In this study, we have investigated the ubiquitin proteasome pathway (MuRF1 and atrogin-1), the autophagy machinery (LC3b) and the matrix metalloproteinase-2 and its inhibitor (MMP-2 and TIMP-2, respectively).

The ubiquitin-dependent proteolysis seems to play a less significant role in the masseter in response to the ICU condition compared with limb muscles. In the masseter, mRNA levels of the atrogenes (MuRF1 and atrogin-1) were up-regulated early, but their respective protein contents were not. In limb muscles, on the other hand, both MuRF1 and atrogin-1 were transcriptionally increased and MuRF1 was up-regulated at the protein level [13], [15]. This reflects muscle-specific differences in MuRF1, but not in atrogin-1 activation. MuRF1 and atrogin-1 are considered indicators of muscle atrophy as their up-regulation is documented in most muscle atrophy conditions and their intrinsic role in muscle atrophy is supported by knockout experiments [39], [40]. The transcription-translation discrepancy for atrogin-1 has been suggested to be due to its long half-life [13], [41]. However, this is not the case for MuRF1 where post-transcriptional regulation appears to play a more important role. Further, MuRF1 has been suggested to repress myosin synthesis at the transcriptional level and to specifically target myosin for degradation via the ubiquitin proteasome pathway [42], facilitating the preferential myosin loss and the subsequent decline in the M/A ratio in ICU patients with CIM. This role of MuRF1 is supported in the masseter by the absence of changes in both MuRF1 protein levels and myosin transcription.

The autophagy degradation pathway seems to be more efficiently regulated in the masseter than in limb muscles. LC3b is a widely used autophagy marker. It is initially expressed as a cytosolic form (LC3b-I) which is subsequently converted into LC3b-II that binds to the autophagosomal membrane and is used as an indicator of autophagosome formation [30]. In the current study, both LC3b forms remained unaffected by the treatment. The stability of the membrane-bound LC3b-II is of special interest here, since it contrasts the up-regulation found in limb muscles after four days of identical ICU condition [13]. Indeed, both autophagosome up- and down-regulation may impair cellular protein balance [43]. The autophagy machinery is involved in protein degradation in skeletal muscles [44] and atrophy conditions, such as starvation [45], denervation [46] and aging [47]. However, the autophagy system has a protective cytotoxic-sequestering function [17], [48] and is required to maintain muscle mass. Further, inhibited autophagy can contribute to myofiber degeneration and weakness characterized by accumulation of dysfunctional mitochondria [44]. The stability of LC3b-II protein levels, and hence autophagosome activity, may be interpreted as an adaptation to the ICU stress and a protective mechanism in the masseter.

Metalloproteinases play an integral role during skeletal muscle atrophy in coordination with MMP negative regulators, TIMPs that prevent the formation of the active proteolytic MMPs [49], [50]. While MMP-2 is considered the primary up-regulated MMP in muscle atrophy [51], it seems to be down-regulated in the masseter after only four days of the experiment. TIMP-2, on the other hand, reported to be down-regulated in many atrophy models, remained unchanged at the protein level throughout experimental period. Although MMP-2 is best known for its degradation of the extracellular matrix proteins in different myopathic conditions, it has also been reported to target sarcomeric proteins, such as troponin I [50] and titin [52], impairing the contractile function of the sarcomere. In addition, MMPs are induced in patients of inflammatory myopathies [53] and inhibited by corticosteroids [49]. Taken together, MMP-2 down-regulation and TIMP-2 steady-state may be involved in better maintenance of the sarcomere stability in the masseter compared with limb muscles and hint towards a lower oxidative stress in the masseter contributing to the spared function in response to the ICU condition.

### Heat shock proteins

Heat shock proteins (HSPs) in cranial nerve innervated muscles seem to be activated more rapidly than in limb muscles. HSP70 increased significantly after 5 days of ICU treatment, while αB-crystallin showed a slight, albeit not statistically significant, early increase. An up-regulation in HSPs protein concentration was also reported in limb muscle ICU response [13], but the onset of these increases was more delayed compared with the masseter, thereby suggesting a faster HSP response in the latter. Heat shock proteins are a cellular defense mechanism involved in muscular tissue remodelling and adaptation, e.g., as a reaction to oxidation, inflammation or energy changes [54]. In addition, HSP70, αB-crystallin and HSP90 also have chaperone-like function contributing to the proper folding of nascent proteins, preventing protein aggregation and stabilizing degrading proteins [55]-[57]. The up-regulation of HSP70 and αB-crystallin and the stability of HSP90 protein levels in the masseter accords with our previous cranial-versus-spinal response to critical illness in a porcine ICU model, where these 3 proteins were induced to a larger extent in the masseter compared with limb muscles [19]. At the same time, HSP activation contrasts the down-regulation of these three particular chaperones described in many unloading models, including hind limb suspension, joint fixing, microgravity and denervation [58]–[62]. The differential HSP response in different models highlights the uniqueness of underlying pathology in our “mechanical silencing” model compared with other atrophy models.

It should also be noted that HSP70 and αB-crystallin have an antiapoptotic function and that they regulate redox signalling of the mitochondria [55], [63], [64]. Moreover, in one study of our porcine critical illness models, HSP70 and αB-crystallin changes correlated with an oxidative stress marker (SOD2) in both cranial- and spinal nerve innervated muscles [19]. HSP90, on the other hand, is involved in the inflammatory signalling via corticosteroid receptors [65], [66]. Based on previous reports and present observations, we may speculate that mitochondrial oxidation and the subsequent inflammatory and apoptotic responses are highly involved in the pathology of CIM.

### Conclusion

In conclusion, the response of cranial nerve innervated masseter to the ICU condition, i.e., sedation, NMB, mechanical ventilation, and immobilization, is clearly different from that of spinal nerve innervated limb muscles. That is, the masseter muscle sustains a high M/A ratio and muscle fiber size for longer periods of exposure to the ICU condition. Compared with previous studies in limb muscles, several mechanisms respond differently in the masseter, e.g., transcriptional regulation of myosin and actin synthesis is maintained with only a transient decline in Akt phosphorylation, absence of activation of the MuRF1, balanced autophagy, inhibited MMP-2 and early activation the protective chaperone machineries. These findings suggest that improved antioxidative profile in the masseter may be a candidate mechanism of preserved masticatory function in CIM. Finally, the experimental rat ICU model closely mimics CIM manifestation in ICU patients supporting its validity and use in future pharmacological interventions studies.
